# Antimicrobial Resistance Profile and Biofilm Formation of *Listeria monocytogenes* Isolated from Meat

**DOI:** 10.3390/antibiotics14050454

**Published:** 2025-04-30

**Authors:** Joana Paiva, Vanessa Silva, Patrícia Poeta, Cristina Saraiva

**Affiliations:** 1 Department of Veterinary Sciences, University of Trás-os-Montes e Alto Douro (UTAD), 5000-801 Vila Real, Portugal; jpaiva@utad.pt (J.P.); ppoeta@utad.pt (P.P.); 2 Animal and Veterinary Research Center (CECAV), University of Trás-os-Montes and Alto Douro (UTAD), 5000-801 Vila Real, Portugal; 3 Microbiology and Antibiotic Resistance Team (MicroART), Department of Veterinary Sciences, University of Trás-os-Montes and Alto Douro (UTAD), 5000-801 Vila Real, Portugal; 4 Department of Genetics and Biotechnology, University of Trás-os-Montes and Alto Douro (UTAD), 5000-801 Vila Real, Portugal; 5 Functional Genomics and Proteomics Unit, University of Trás-os-Montes and Alto Douro (UTAD), 5000-801 Vila Real, Portugal; 6 Associated Laboratory for Green Chemistry of the Network of Chemistry and Technology (LAQV-REQUIMTE), Department of Chemistry, NOVA School of Science and Technology, Nova University of Lisbon, 1099-085 Caparica, Portugal; 7 Associate Laboratory for Animal and Veterinary Sciences (AL4AnimalS), 5000-801 Vila Real, Portugal

**Keywords:** *Listeria monocytogenes*, antimicrobial resistance, biofilm formation, meat, genotypic resistance

## Abstract

Introduction: *Listeria monocytogenes* is the causative agent of listeriosis, a serious infectious disease with one of the highest case fatality rates among foodborne diseases affecting humans. Objectives: This study investigated the prevalence, antimicrobial resistance pattern and biofilm production capacity of *L. monocytogenes* isolated in meats. Materials: A total of 75 samples were analyzed, including fresh meats and meat preparations, in Northern Portugal. Methods: The strains were identified using morphological and molecular methods. Antimicrobial resistance was determined using the Kirby–Bauer disk diffusion method, against a panel of 12 antibiotics and the presence of the respective antimicrobial resistance genes was investigated by polymerase chain reaction (PCR). The ability to form biofilms was evaluated by the microtiter biofilm assay. Results: The overall prevalence of *L. monocytogenes* among screened samples was 17.33%. The isolates were resistant to trimethoprim-sulfamethoxazole (85.71%), ciprofloxacin (38.10%), meropenem (33.33%), tetracycline and erythromycin (28.57%), rifampicin (23.81%), and kanamycin (14.29%). Six isolates (28.57%) exhibited a multidrug-resistance profile. All strains showed positive result for the virulence gene specific to listeriolysin O (*hly*A). In the genotypic resistance analysis of the strains, the genes identified were *tet*K (23.81%), *aad*A, *tet*L, *bla*_OXA-48_ (14.29%), *erm*C, and *msr(A/B)* (4.76%). All isolates had the ability to form biofilms, with no significant differences in biofilm biomass production at 24 h and 48 h. Some of these strains showed a high capacity for biofilm production. Conclusions: These findings raise public health concerns due to resistance to first-line antibiotics and the biofilm-forming capacity of these isolates, which pose risks to the food industry. Enhanced monitoring and surveillance are essential to guide public health strategies in order to mitigate the threat posed by *L. monocytogenes* in food.

## 1. Introduction

*Listeria monocytogenes* is a foodborne pathogen commonly found associated with human and animal listeriosis [1,2]. This bacterium, which was first identified as a human pathogen in 1929, was only recognized as a threat to public health in the early 1980s, when it was found to be transmitted through food. Nowadays, almost all cases of listeriosis are recognized as foodborne and serious attention has been given to the study of this pathogen [2,3]. *L. monocytogenes* is an ubiquitous, facultative Gram-positive intracellular pathogen. As these bacteria are resistant to large variations in temperature (−0.4–45 °C), pH (4.1–9.6) and salt concentrations (up to 20% *w*/*v*), they have the ability to survive and multiply under extreme environmental conditions. These characteristics contribute to their versatility to grow in food-processing environments, posing a significant challenge for the food industry [4,5,6]. This pathogen is widely prevalent in the environment and responsible for several outbreaks of listeriosis linked to the consumption of contaminated products [1,5]. *L. monocytogenes* is prevalent in the food industry, particularly in ready-to-eat (RTE) foods, such as meats, fish, dairy products, and vegetables [7,8]. Due to its chemical and physical properties, fresh meat and meat products comprise an extremely favorable environment for the development and reproduction of microorganisms, whether spoilage or pathogenic bacteria. These foodstuffs are linked to significant listeriosis outbreaks, being considered the main source of *L. monocytogenes* infections in humans [1,3,9].

Listeriosis can cause either non-invasive febrile gastroenteritis with flu-like symptoms in healthy people or serious invasive illness with meningitis, septicemia and encephalitis in newborns, immunocompromised, and elderly people, and abortion in pregnant women, with a mortality that ranges from 20 to 40% [1,3,10]. According to the 2024 One Health zoonoses report by the European Food Safety Authority (EFSA), *L. monocytogenes* infections were among the most reported zoonotic agents in humans. Additionally, listeriosis was the most severe zoonotic disease, with the highest percentage of hospitalizations among confirmed cases (96.5%) and the highest case fatality rates (19.7%) [7].

In recent decades, there has been a rising concern over antimicrobial resistance in *L. monocytogenes*. Several reports indicate an increased resistance to a variety of current administered antibiotics, some of them considered last resource antibiotics [2,11]. As with some other bacteria, resistance properties can be spread among *L. monocytogenes* by gene transfer [12]. Spread of antimicrobial resistance between countries and continents has been increasing because of the global trade and travel around the world [3,6]. The emergence of resisting clones is linked to the selective pressure of antimicrobial agents in humans, animals, and food production, with global implications in public health. The widespread, excessive, and incorrect use of antibiotics contributes to bacterial resistance, making the treatment more complex [1,3,4].

Several adaptations strategies, including attachment to surfaces, increased resistance to environmental stresses and the ability to form biofilms, are found in *L. monocytogenes* to thrive in various adverse and stress conditions [5,13]. Biofilms may be defined as aggregates of bacterial cells that form communities surrounded by an extracellular matrix [14,15]. *L. monocytogenes* has the ability to form biofilms on food-processing contact surfaces, allowing it to persist over time and posing a major challenge to the food industry. These biofilms can remain viable for months or even years, enabling recurrent contamination of food products. The persistence of *L. monocytogenes* in food processing environments has been closely associated with its biofilm-forming capacity, and was recently highlighted by the EFSA as one of the main bacterial hazards in food and feed processing facilities [16,17]. Additionally, these structures exhibit a greater resistance to antimicrobial and disinfectant agents [18]. More importantly, this affects the virulence potential, pathogenicity, survival capability, and therapeutic potential of this pathogen [4]. Therefore, control of this pathogen in food is a significant challenge [13].

The aim of this study was to evaluate the prevalence of *L. monocytogenes* isolated from fresh meat and meat products, from the North of Portugal. Additionally, this study aimed to characterize the isolates in terms antimicrobial resistance and biofilm production capacity.

## 2. Results

### 2.1. Prevalence and Virulence of L. monocytogenes Strains

A total of twenty one *L. monocytogenes* isolates were obtained from 75 fresh meat and meat preparations, corresponding to 12 positive meat samples, which results in a prevalence of 16%, as primarily confirmed by the characteristics of this pathogen. To confirm the isolates species, 16S rDNA gene was amplified by PCR and then sequenced. Table 1 shows the phenotypical and genotypical characterization of the strains. All the isolates tested positive for the virulence gene specific to listeriolysin O (*hly*A) in the PCR assay.

### 2.2. Susceptibility to Antibiotics in L. monocytogenes Strains

**Figure 1:**
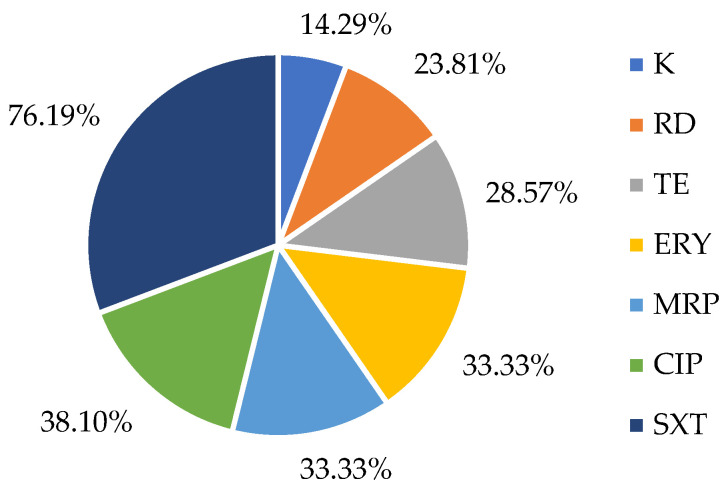
Percentage of resistance to different antibiotics tested among *L. monocytogenes* isolates. TE: tetracycline; RD: rifampicin; CIP: ciprofloxacin; K: kanamycin; ERY: erythromycin; MRP: meropenem; SXT: trimethoprim/sulfamethoxazole.

Among the antibiotics tested, trimethoprim-sulfamethoxazole exhibited the highest prevalence of resistance (76.19%), followed by meropenem (33.33%), tetracycline and erythromycin (28.57%), rifampicin (23.81%) and kanamycin (14.89%), as seen in Figure 1. Resistance to ampicillin, chloramphenicol, gentamicin, linezolid or vancomycin was not detected.

In our study, one *L. monocytogenes* isolate for gentamicin and six isolates for vancomycin were classified as ‘Susceptible, increased exposure’, highlighting the potential need for dosage adjustment when treating infections caused by these strains. According to the EUCAST classification [19], this category indicates that these antibiotics may remain effective, provided that higher doses or optimized exposure are applied.

Notably, 28.57% of the *L. monocytogenes* strains (n = 6) exhibited multidrug resistance (MDR), defined as resistance to at least three different classes of antimicrobials [20]. The multidrug-resistance phenotype of these strains is detailed in Table 1. Among the six MDR strains, one was resistant to seven classes of antimicrobials (RD-CIP-K-E-TE-MRP-SXT), four were resistant to six classes (RD-CIP-K-E-MRP-SXT and RD-CIP-E-TE-MRP-SXT), and one was resistant to four classes (CIP-E-MRP-SXT). Additionally, all six strains shared resistance to ciprofloxacin, erythromycin, meropenem, and trimethoprim-sulfamethoxazole.

### 2.3. Antimicrobial Resistance Genes Presence in L. monocytogenes Strains

Only seven *L. monocytogenes* isolates exhibited the presence of various antimicrobial resistance gene sequences, as demonstrated in Table 1. The predominant resistance gene was associated with tetracycline, with five out of 21 (23.81%) isolates testing positive for at least one of the relevant genes. The most frequent gene detected among the 21 isolates was *tet*K (23.81%), followed by *tet*L, *aad*A and *bla*_OXA-48_ (14.29%) and *erm*C and *msr(A/B)* (4.76%). Conversely, other resistance genes (*tet*A, *tet*M, *aac*(6′)-Ie-*aph*(2″)-Ia, *erm*A, *bla*_OXA_ and *sul*1) were absent in all tested *L. monocytogenes* isolates from the meat products.

The *aad*A gene, which is commonly associated with aminoglycoside resistance, was identified in 100% of kanamycin-resistant isolates. Additionally, the *bla*_OXA-48_ gene, known for its role in carbapenem resistance, was detected in 42.86% of meropenem-resistant isolates. Among tetracycline-resistant strains, 33.34% and 16.67% harbored *tet*L and *tet*K genes, respectively. Erythromycin-resistant isolates carried *erm*C and *msr*(A/B) genes in 14.29% of the cases, both of which are associated with macrolide resistance.

### 2.4. Biofilm Formation of the Isolates

**Figure 2:**
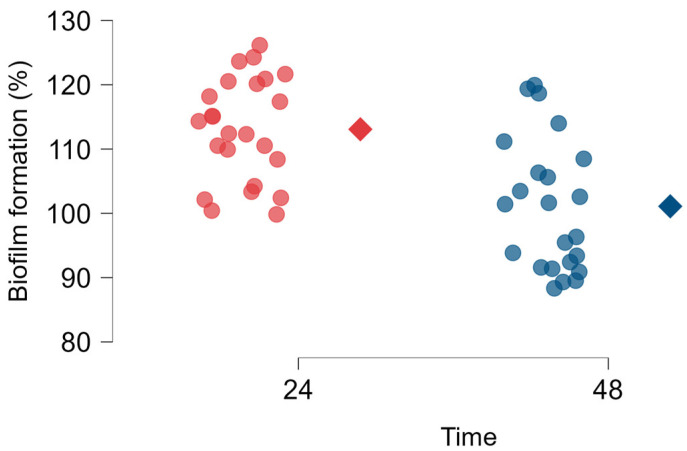
Biofilm formation capacity (expressed as % in comparison to the reference strain) of *L. monocytogenes* strains isolated from different meats products. Red and blue dots represent the mean biofilm biomass formed in independent tests of the individual isolates at 24 h and 48 h, respectively. The red and blue diamond shape symbols indicate the group average biofilm biomass at 24 h and 48 h, respectively. Statistical significance was determined using one-way ANOVA followed by Tukey’s multiple comparison test (*p* < 0.01).

All strains isolated from different samples produced biofilm at 24 h and 48 h. The results were normalized against *L. monocytogenes* ATCC^®^ 7973 so that the comparison of results could be more consistent. Figure 2 shows the biofilm formation of each isolate at 24 h and at 48 h. *L. monocytogenes* strains at 24 h were the ones that produced the most biofilm, with a percentage mean of biofilm formation of 113.08 ± 7.88, which was significantly higher (*p* < 0.001) than the biofilms at 48 h, with a percentage mean of biofilm formation of 103.72 ± 16.01. Based on our results, the biofilm production capacity of *L. monocytogenes* at 24 h and at 48 h was, on average, higher than that of the reference strain. However, not all isolates showed biofilm formation levels above the reference strain, particularly at 48 h. The strains isolated in this work include highly productive biofilm strains, as some produced significantly more biofilm than the reference strain.

## 3. Discussion

Our research has shown that the prevalence in Northern Portugal, is 17.33% in fresh meat and meat products investigated, obtained in retail markets. In 2022, a separate investigation assessing the prevalence of *L. monocytogenes* in twenty chicken minced meat samples from butcher’ shops from the North-Western Iberian Peninsula, demonstrated higher results, with a prevalence of 70% [21]. In a study conducted in Portugal by Henriques et al. (2017), *L. monocytogenes* was detected in 10 out of 100 retail ready-to-eat (RTE) samples (10%) [22]. Research conducted under similar conditions in different countries, in meat and meat products from retail markets, show a maximum prevalence of *L. monocytogenes* of 15.3% [5,23,24]. In the literature, similar studies reported prevalences generally lower than those observed in this study. For example, Yushina et al. (2022) recorded a prevalence of 8.8% in raw meat from different species in Russia [5]. Similarly, Cavalcanti et al. (2022), after analyzing 29 studies conducted between 2009 and 2019, found an average prevalence of 14% in meat in Brazil [24]. However, there is a lack of data on the prevalence and antibiotic resistance profiles of strains isolated from meat products in Portugal. The observed prevalence of *L. monocytogenes* suggests a potential need for increased surveillance and control measures to prevent further contamination and mitigate public health risks.

Regarding antimicrobial resistance, all isolates were susceptible to ampicillin, chloramphenicol, gentamicin, linezolid and vancomycin. Linezolid and vancomycin are critical last-resort antibiotics, strictly reserved for use in hospital settings. The absence of resistant isolates in this study is highly significant, as it highlights the importance of preserving the efficacy of these critical drugs and ensuring their continued effectiveness against *L. monocytogenes*. A high rate of resistance to trimethoprim-sulfamethoxazole (SXT) was also found in *L. monocytogenes* isolates obtained from meat samples in Morocco [25]. The high resistance rate to trimethoprim-sulfamethoxazole in *L. monocytogenes* isolated from meat can be attributed to various factors, including the excessive use of antibiotics in animal production and the transfer of resistance genes between bacteria. A recent study conducted in Romania analyzed 26 *L. monocytogenes* strains isolated from ready-to-eat products and found a prevalence of SXT resistance of 26.92%. The authors emphasize the importance of continuous surveillance to monitor antimicrobial resistance and guide public health strategies [26]. A recent study reviewed the antimicrobial resistance of *L. monocytogenes* isolated from meat and meat products. Although most strains are sensitive to antibiotics, resistance to fluoroquinolones like ciprofloxacin has been reported in some foodborne isolates. In line with these findings, our study reveals a resistance rate of 38.1% to ciprofloxacin among *L. monocytogenes* isolates from meat products, which highlights a concerning trend. Resistance can be attributed to the excessive use of antibiotics in veterinary medicine and agriculture, exerting selective pressure on bacteria, which promotes the emergence of resistant strains [6]. Other similar studies reported higher resistance rates to chloramphenicol, erythromycin, gentamicin and vancomycin and a lower percentage of resistance to ciprofloxacin [25,27]. In 2020, Matle et al. published a review about antimicrobial resistance in *L. monocytogenes* from meat and meat products, where they found resistance against most of the antimicrobials that we tested [6]. However, the percentage of resistance detected in our study is considerably higher compared to that reported in other studies. In a different study (published in 2023) from the European Union, namely Poland, out of 153 *L. monocytogenes* samples originating from meat products and processing environments, almost all (n = 143, 93.5%) collected bacteria were susceptible to all tested antibiotics (namely, ampicillin, chloramphenicol, erythromycin, gentamycin, penicillin, streptomycin, sulfamethoxazole-trimethoprim, tetracycline and vancomycin). What is more, none of the isolates was resistant to more than one antibiotic. The only antibiotic to which collected isolates showed reduced susceptibility was ciprofloxacin, to which ten isolates (6.5%) were classified as intermediate [28]. Additionally, in a study from Japan, published in 2019, all 85 tested *L. monocytogenes* isolates originating from chicken meat were susceptible to ampicillin, gentamycin, erythromycin, vancomycin and sulfamethoxaloze-trimethoprim and just one case of resistance to linezolid [1]. Amoxicillin, ampicillin and penicillin are among the beta-lactam antibiotics, known for their highly inhibitory effect on Gram-positive bacteria, including *L. monocytogenes*. For this reason, they are frequently used in the treatment of listeriosis [3,4]. Therefore, we would expect a higher percentage of resistance against ampicillin, which recorded 100% susceptibility in our *L. monocytogenes* isolates. In our study we found 28.57% isolates that showed a multidrug-resistance profile. These results are in accordance to the paper by Tayeb et al. (2023), which had 22.97% of multidrug-resistance in meat and meat products [29]. Nevertheless, Maung et al. (2019) reported 55.1% of multidrug-resistance strains in 135 raw chicken meat samples, a value two times higher than ours [1]. Interestingly, the antibiotics with the highest resistance rates were oxacillin, cefoxitin, and fosfomycin [1].

Among all resistance genes that we tested, the ones that confer resistance to tetracycline, *tet*L and *tet*K, were the most frequent ones (33.34% and 16.67%, respectively). Tetracycline is one of the most used antibiotics in animal production, which can justify the high prevalence of resistance genes for this antibiotic in strains isolated from meat samples [30,31]. Indeed, the percentages refer to the resistance genes detected within the phenotypically resistant strains to tetracycline. However, it is important to note that despite the absence of specific tetracycline resistance genes in a portion of the tetracycline-resistant *L. monocytogenes* isolates, this does not exclude the possibility of other resistance mechanisms at play. *L. monocytogenes* has not been as extensively studied in terms of its resistance mechanisms as some other bacterial species, and thus, there may be additional, yet unidentified or unreported mechanisms contributing to the resistance phenotype. It is noteworthy that the majority of resistances in our study were found in strains isolated from minced meat samples. In fact, the processing and handling of ground beef not only increases its microbial load but also contribute to the dissemination of antibiotic-resistant bacteria. Increased manipulation can introduce and spread resistant bacteria, resulting in a higher prevalence of resistant strains in minced meat.

Biofilms are aggregates of microbial cells that are interconnected and adhere tightly to each other or to a surface. Enveloped in an extracellular multicellular matrix, they play an important role in microbial survival in challenging environments by providing phenotypic adaptability and ecological advantages. *L. monocytogenes* is resilient and can colonize and survive in food processing facilities. The ability to form biofilms promotes its growth and proliferation in harsh environments [4,32]. In our study, the evaluation of 21 strains of *L. monocytogenes* from fresh meats and meat products demonstrated the potential to form 24 h and 48 h biofilms, as depicted in Figure 2. There was a highly statistically significant difference (*p* < 0.001) between the biofilm biomass at 24 h and 48 h, with the average being lower at 48 h. In any case, some strains show an increase or decrease in biofilm biomass between 24 h and 48 h. These variations may be due to factors such as environmental conditions, nutrient availability, and the expression of biofilm-associated genes [33,34]. However, we cannot rule out possible technical errors, such as the rupture and loss of biofilm pieces during the washing procedure, as the adhesion capacity of different species to substrates differs [33,35]. It would be expected that during the first three days, the biofilm growth rate would be higher, as it corresponds to the biofilm maturation period, growing at a lower rate on the fourth and fifth days. From the sixth day onwards, with the dispersion of biofilm cells, a decrease in its biomass is natural [36]. All strains had a strong biofilm-forming ability, and our biofilm production was much higher when compared to other similar studies, with a high percentage of highly biofilm-producing strains. The fact that the strains show high biofilm production constitutes a public health problem since the formation of biofilms represents a major risk in the food industry and may be responsible for economic losses. Biofilms are forms of survival for these microorganisms and make their infections extremely difficult to treat, due to the immune response they provoke and the resistance to antimicrobials and other agents [37]. Several studies have evaluated biofilm formation. In 2022, Ciccio et al. studied 28 strains from meat products (n = 20) and meat processing environments (n = 8) to evaluate their biofilm-production capability. All tested *L. monocytogenes* strains were biofilm producers and the isolates exhibited varying levels of biofilm formation, from weak to moderate or strong production. Interestingly, 57% (n = 16) of the isolates from meat products were classified as moderate or strong biofilm producers [38]. In a study conducted by Doijad et al. (2015), the biofilm-forming ability of *L. monocytogenes* strains isolated from meat was also evaluated [18]. All strains were biofilm producers, with 85.71% exhibiting low biofilm production, 14.29% showing moderate biofilm production, and none classified as high biofilm producers [18].

## 4. Materials and Methods

### 4.1. Sample Collection and Bacterial Isolates

Seventy-five meat samples were collected from hypermarkets and small local shops, including fresh meats, meat preparations (such as meatballs, hamburgers, fresh sausages, meat breading, and skewers), and meat-based products (e.g., alheira—a Portuguese sausage made of meat, typically pork, mixed with bread and garlic, and moura—a type of traditional Portuguese sausage made with pork and blood), in 2022. The distribution of samples was as follows: 30 samples (40%) of minced meat, 9 samples (12%) of meatballs and hamburgers, 9 samples (12%) of meat skewers, 6 samples (8%) of breaded meat, 6 samples (8%) of fresh sausages, and 15 samples (20%) of meat-based products (“alheira” and “moura”). All samples were transported in ice boxes to the laboratory within 2 h after sampling and analyzed immediately.

### 4.2. Detection and Isolation of L. monocytogenes

The bacterial isolation protocol was performed according to the horizontal detection and counting method for *L. monocytogenes*, following the EN ISO 11290-1:2017 guidelines [39]. Briefly, 25 g of each sample was aseptically homogenized with 225 mL of selective half Fraser broth (Oxoid, Thermo Fisher Scientific, Basingstoke, UK) and incubated at 30 °C for 24 h as primary enrichment. A secondary enrichment was performed, which consisted of adding 0.1 mL of the broth culture to 10 mL of full-strength Fraser broth (Oxoid, Thermo Fisher Scientific, Basingstoke, UK) and incubation at 37 °C for 24 h. After incubation, a loopful from both enrichment cultures was plated onto CHROMagar™ Listeria (CHROMagar, Paris, France) and in Oxford agar (Oxoid, Thermo Fisher Scientific, Basingstoke, UK), and incubated at 37 °C for 24–48 h. Presumptive colonies of *L. monocytogenes* were identified by blue colonies with white halos on CHROMagar and brown colonies with black halos on Oxford agar. These colonies were then streaked onto trypticase soy agar supplemented with 0.6% yeast extract (TSA-YE) (Liofilchem, Roseto degli Abruzzi, Italy) and incubated at 37 °C for 18–24 h. The colonies developed on TSA-YE media were confirmed as *L. monocytogenes* through biochemical tests. All isolates were catalase-positive and oxidase-negative. In carbohydrate fermentation tests, isolates fermented L-rhamnose (positive) but did not ferment D-xylose (negative), consistent with the typical biochemical profile of *L. monocytogenes*.

### 4.3. Antimicrobial Susceptibility Testing

After isolation, the frozen *L. monocytogenes* strains were thawed and then plated onto Brain Heart Infusion agar, before being incubated at 37 °C for 24 h. They were characterized according to their antibiotic resistance profiles using the Kirby–Bauer disk-diffusion method against 12 antibiotics (Oxoid, Thermo Fisher Scientific, Basingstoke, UK), following the standard protocol recommended by the European Committee on Antimicrobial Susceptibility Testing [19]. Briefly, Briefly, 5–6 colonies from overnight cultures were suspended in 1 mL of 0.9% NaCl solution, and the turbidity was adjusted to a 0.5 McFarland standard. Afterwards, the suspension was inoculated onto Mueller–Hinton agar. Antibiotic discs were placed on the agar surface at intervals of 3 cm, each disc containing a specific antibiotic concentration: ampicillin (AMP, 10 µg), chloramphenicol (C, 30 µg), ciprofloxacin (CIP, 5 µg), erythromycin (ERY, 15 µg), gentamicin (CN, 10 µg), kanamycin (K, 30 µg), linezolid (LNZ, 30 µg), meropenem (MRP, 10 µg), rifampicin (RD, 5 µg), tetracycline (TE, 30 µg), trimethoprim/sulfamethoxazole (STX, 1.25/23.75 µg) and vancomycin (VA, 30 µg). Subsequently, the plates were incubated at 37 °C for 24 h. The diameter of the inhibition zones was measured to the nearest millimeter. In cases where the EUCAST guidelines did not provide resistance criteria for *Listeria* spp., any missing breakpoints were supplemented with those recommended for Staphylococcus aureus and Enterococcus spp. according to CLSI standards. Classification of isolates as resistant (R), susceptible, increased exposure (I), or susceptible, standard dosing regimen (S) to a particular antibiotic was based on the result obtained using standard reference values according to the EUCAST guidelines [19]. A stock culture of *L. monocytogenes* strain, ATCC^®^ 7973, was used as a reference strain.

### 4.4. DNA Extraction

The extraction of genomic DNA was performed as previously described [40]. Briefly, two colonies of fresh cultures from each isolate were suspended in 45 µL of Milli-Q water. Five microliters of lysostaphin (1 mg/mL) were added, and the samples were incubated for 10 min at 37 °C. Then, 150 µL of Tris-HCl (0.1 M), 45 µL of Milli-Q water and 5 µL of proteinase K (2 mg/mL) were added, and the samples were incubated at 67 °C for 10 min. Lastly, samples were boiled for 5 min at 100 °C. DNA was quantified using the ND-100 Spectrophotometer (NanoDrop^®^ spectrophotometer, Thermo Fisher Scientific, Waltham, MA, USA).

### 4.5. Characterization of Resistance Genes and Detection of Virulence Genes

The presence of antibiotic resistance genes was studied by PCR and sequencing using specific primers. Based on the phenotypic resistance results, the isolates were screened for the corresponding antimicrobial resistance genes. The following resistance genes were investigated: tetA, tetK, tetL and tetM (tetracycline resistance); aac(6′)-Ie-aph(2″)-Ia and aadA (kanamycin resistance); ermA, ermC and msr(A/B) (erythromycin resistance); bla_OXA-48_ and bla_OXA_ (meropenem resistance); and sul1 (resistance to trimethoprim/sulfamethoxazole) were screened by PCR. The presence of virulence factor gene hlyA (alpha hemolysin) was determined in all isolates using PCR. The amplification products were confirmed by agarose gel electrophoresis (1.5% *w*/*v*), stained with ethidium bromide, and visualized under UV light. Amplicon sizes were compared with a DNA ladder to verify the specificity of the PCR amplification. The positive and negative controls used belong to the strain collection of the University of Trás-os-Montes and Alto Douro.

### 4.6. Assessment of Biofilm-Forming Ability

The biofilm-forming capacity of the isolates was determined after 24 h and 48 h. The biofilm-producing *L. monocytogenes* strains were detected by the microtiter plate-based method (MM). This method was used to assess the biofilm-forming capabilities of the strains. The MM is a qualitative assessment of biofilm formation and was performed as previously described with some modifications [41]. Two colonies were transferred from recently grown cultures to tubes containing 3 mL of Tryptic Soy Broth (TSB; Liofilchem, Teramo, Italy) and then placed in an incubator at 37 °C for approximately 16 h with continuous shaking at 150 rpm (ES-20 Shaker-Incubator, BioSan, Riga, Latvia). Following the incubation period, the bacterial suspension was adjusted to an optical density equivalent to 2 × 10^6^ colony-forming units (Biochrom; EZ Read 800 Plus; Cambridge, UK). Subsequently, 200 µL of the bacterial suspension from different isolates was added to each well of a 96-well flat-bottom microplate for 24 h and 48 h (Orange Scientific, Braine-l’Alleud, Belgium). A negative control consisting of fresh medium without any bacterial inoculum was included. The microplates were then incubated at 37 °C for 24 h without shaking. The medium in the 48 h microplate was carefully replaced with fresh medium at 24 h. Each experiment was performed in triplicate and had seven technical replicates.

The quantification of biofilm mass was performed using the Crystal Violet (CV) staining method following the protocol described by Peeters et al. (2008), with some modifications [42]. After the incubation period, the plates were washed twice with 200 µL of distilled water to eliminate non-adherent bacterial cells and were then allowed to air dry at room temperature for approximately 2 h. Subsequently, 100 µL of methanol (Fisher Scientific, Leicestershire, UK) was added to each well and incubated for 15 min to fix the microbial biofilm. The methanol was removed, the plates were left to dry for 10 min at room temperature, and 100 µL of CV at 1% (*v*/*v*) (Acros Organics, NJ, USA) was added to each well. The CV solution was then discarded, and the plates were washed twice with distilled water to remove excess dye. Following this, 100 µL of 33% (*v*/*v*) acetic acid was added to each well to solubilize the CV, and the absorbance was measured at 570 nm using the microplate reader BioTek ELx808U (BioTek, Winooski, VT, USA) [42].

### 4.7. Statystical Analysis

Descriptive statistics of data are presented as the mean and standard deviation when appropriate. Additionally, *t*-test analysis and ANOVA followed by Tukey’s post-hoc test, with a significance level of *p* ≤ 0.05, were used. All statistical analysis was performed using SPSS (version 30.0.0, IBM SPSS Statistics, Chicago, IL, USA).

## 5. Conclusions

A moderate frequency of *L. monocytogenes* was found among fresh meat, meat products and meat preparations, available for human consumption. Six strains were multidrug-resistant with a diversity of antimicrobial resistance. All strains were able to form biofilms. These results underscore the role of livestock animals as reservoirs of antimicrobial-resistant *L. monocytogenes* and resistance genes, posing a significant threat to public health due to resistance against antimicrobials essential in human medicine. Further research is required to fully elucidate the complex mechanisms underlying antimicrobial resistance in *L. monocytogenes* and to identify potential novel resistance factors. The ability of these strains to form biofilms further exacerbates contamination risks in food processing environments. To mitigate these risks, frequent monitoring of livestock strains is essential to track the evolution and dissemination of resistance genes and their zoonotic potential. Additionally, strategies such as educating livestock producers, restricting antibiotic use, and implementing stricter antimicrobial prescription legislation in Portugal are critical to combating antimicrobial resistance in the meat sector.

## Figures and Tables

**Table 1:** Phenotypic and genotypic antimicrobial resistance, virulence and sources of *L. monocytogenes* strains isolated from meat samples.

	Antimicrobial Resistance			
Isolate Code	Phenotype	Genotype	Virulence Gene Detected	Source
JP01	-	-	*hly*A	*Alheira*
JP02	SXT	-	*hly*A	*Alheira*
JP03	TE	-	*hly*A	Meat skewer
JP04	TE	-	*hly*A	Meat skewer
JP05	RD-CIP-K-ERY-MRP-SXT	*aad*A, *tet*K	*hly*A	Minced meat (bovine)
JP06	RD-CIP-K-ERY-MRP-SXT	*aad*A, *tet*K	*hly*A	Minced meat (bovine)
JP07	RD-CIP-ERY-TE-MRP-SXT	*erm*C, *tet*L	*hly*A	Minced meat (poultry)
JP08	RD-CIP-ERY-TE-MRP-SXT	*bla*_OXA-48_, *tet*K, *tet*L	*hly*A	Minced meat (poultry)
JP09	CIP-ERY-MRP-SXT	*bla*_OXA-48_, *tet*K, *tet*L	*hly*A	Minced meat (poultry)
JP10	RD-CIP-K-ERY-TE-MRP-SXT	*aad*A, *bla*_OXA-48_	*hly*A	Minced meat (poultry)
JP11	-	-	*hly*A	Breaded meat
JP12	SXT	-	*hly*A	Fresh sausage (swine)
JP13	CIP-SXT	-	*hly*A	Fresh sausage (swine)
JP14	-	-	*hly*A	Minced meat (bovine)
JP15	TE-SXT	-	*hly*A	Meat skewer
JP16	SXT	-	*hly*A	Meat skewer
JP17	SXT	-	*hly*A	Meat skewer
JP18	MRP-SXT	-	*hly*A	Minced meat (poultry)
JP19	CIP-SXT	-	*hly*A	Minced meat (poultry)
JP20	ERY-SXT	*msr*(A/B)	*hly*A	*Alheira*
JP21	SXT	-	*hly*A	*Alheira*

TE: tetracycline; RD: rifampicin; CIP: ciprofloxacin; K: kanamycin; ERY: erythromycin; MRP: meropenem; SXT: trimethoprim/sulfamethoxazole; *aad*A: encodes a streptomycin/spectinomycin aminoglycoside adenylyltransferase (AAD) enzyme; *erm*C: encodes the rRNA adenine N-6-methyltransferase; *tet* genes encode a tetracycline efflux pump; *bla*_OXA-48_: encodes a carbapenem-hydrolyzing class D β-lactamase; *msr*(A/B): encodes the peptide methionine sulfoxide reductase.

## Data Availability

The original contributions presented in the study are included in the article, further inquiries can be directed to the corresponding author.
